# A novel *SLC5A2* heterozygous variant in a family with familial renal glucosuria

**DOI:** 10.1038/s41439-022-00221-w

**Published:** 2022-12-01

**Authors:** Maho Hatano, Tomohiro Udagawa, Toru Kanamori, Akito Sutani, Takayasu Mori, Eisei Sohara, Shinichi Uchida, Tomohiro Morio, Masato Nishioka

**Affiliations:** 1 Department of Pediatrics, Kawaguchi Municipal Medical Center, 180, Nishiaraijuku, Kawaguchi, 333-0833 Saitama, Japan; 2 Department of Ped iatrics and Developmental Biology, Graduate School of Medical and Dental Sciences, Tokyo Medical and Dental University (TMDU), 1-5-45, Yushima, Bunkyo-ku, 113-8519 Tokyo, Japan; 3 Department of Nephrology, Graduate School of Medical and Dental Sciences, Tokyo Medical and Dental University (TMDU), 1-5-45, Yushima, Bunkyo-ku, 113-8519 Tokyo, Japan

**Keywords:** Rare variants, Paediatric kidney disease, Genetic predisposition to disease, Disease genetics, Urinary tract infection

## Abstract

Familial renal glucosuria (FRG) is characterized by persistent glucosuria despite normal blood glucose levels in the absence of overt tubular dysfunction. SGLT2 is a sodium-glucose cotransporter expressed in the proximal tubule; loss-of-function variants in *SLC5A2* are the primary cause of FRG. Heterozygous variants have rarely been reported in Japanese individuals. Here, we identified a novel *SLC5A2* heterozygous variant, c.1348G>T: p.Gly450Trp, in a Japanese family comprising two children and their father.

Familial renal glucosuria (FRG) is characterized by persistent glucosuria despite normal blood glucose levels in the absence of overt tubular dysfunction^1^. Mutations in sodium/glucose cotransporter 2 (SGLT2, *SLC5A2*, OMIM: 182381) have been identified and were recently reported to be involved in FRG. SGLT2 is critical for glucose reabsorption in the proximal convoluted tubules^2^. The *SLC5A2* gene [7.7 kb, on chromosome 16p11.2] contains 14 exons and encodes 672 amino acids. FRG is generally caused by homozygous or compound heterozygous variants of *SLC5A2*^3,4^. Here, we report a case of a Japanese family with a novel heterozygous variant (NM_003041.4: c.1348G>T: p.Gly450Trp) of *SLC5A2*.

The probands were two Japanese sisters: a 9-year-old girl (Patient 1) and a 7-year-old girl (Patient 2). They underwent a health checkup and exhibited urinary glucose positivity. Patient 1 presented with persistent urinary glucose excretion and was diagnosed with renal glucosuria when she was five years old. However, her condition was not critical. Family history (Fig. 1a) revealed that her father and her great-grandfather also exhibited urinary glucose excretion but had not been diagnosed with diabetes or renal disease. Her mother did not present with glucosuria or hyperglycemia. The medical and developmental histories of both patients were insignificant. They exhibited normal body mass index (BMI) and did not have cataracts or a Kayser-Fleischer ring. Clinical findings (Table 1) showed no signs of hyperglycemia or elevated levels of HbA1c, creatinine, or liver enzymes. The probands were diagnosed with renal glucosuria since there were few characteristic findings suggestive of Fanconi syndrome, Lowe syndrome, or Wilson disease.

**Fig. 1:**
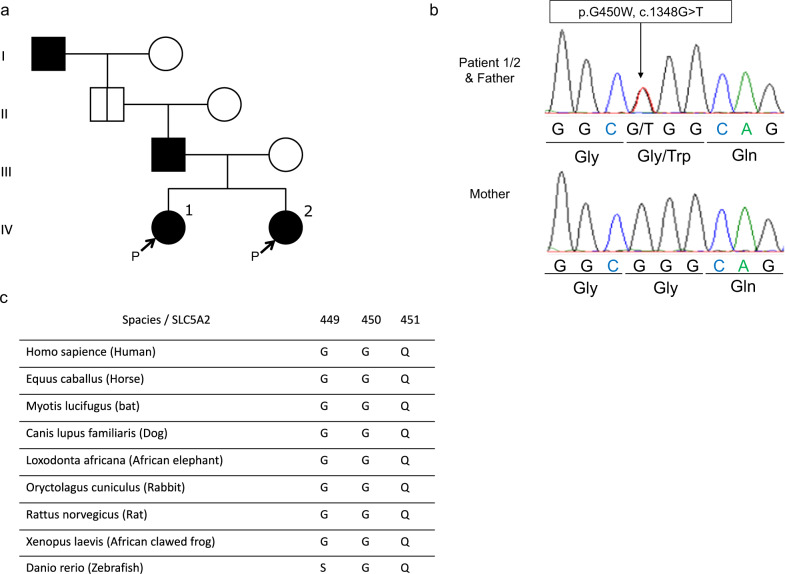
Heterozygous mutation in *SLC5A2* with familial renal glucosyuria. **a** Family pedigree. Patients 1 and 2 and their father and grandfather were diagnosed with FRG. The grandfather’s detailed medical history was unknown, and he was an *SLC5A2* carrier. b,c. Mutation analysis in family members affected by FRG. **b** Genomic DNA sequence analysis. The G → T transition at *SLC5A2* cDNA position 1348 results in a glycine to tryptophan substitution at position 450 of the SLC5A2 protein (Gly450Trp). The variant was carried in a heterozygous state by Patient 1, Patient 2, and their father. The unaffected mother displayed a wild-type (WT) sequence. **c** Protein alignment. The G450 residue (highlighted with red color) is conserved among the human SGLT subtypes and across SGLTs of various other species.

**Table 1:** Clinical features of family members.

	Patient1	patient2	father
Age(years)	9	7	47
Gender	Female	Female	Male
Height(cm)	124	123	ND
Weight(kg)	24.9	25.6	ND
*Blood biochemistry*			
Plasma glucose(mg/dl)	101	94	ND
Total Protein(g/dl)	7.2	7.1	ND
Albumin(g/dl)	4.5	4.5	ND
Creatinine(mg/dl)	0.44	0.4	ND
eGFR	97.17	105.51	ND
Sodium(mmol/L)	140	141	ND
Potassium(mmol/L)	4.1	3.9	ND
Chloride(mmol/L)	105	106	ND
AST(U/L)	28	28	ND
ALT(U/L)	33	24	ND
HbA1c(%)	5.4	5.2	ND
*Urinary analysis*			
Urine glucose	2+	1+	1 + −4+
Urine Glucose (mg/dL)	95	51	ND
Urine glucose/Cre (g/gCre)	1.35	0.5	ND
Urine protein (mg/dL)	5	6	ND
Urine TP/Cre (g/gCr)	0.05	0.09	ND
Urine RBC (/HPF)	<1	<1	ND
Urine WBC (/HPF)	<1	<1	ND
pH	6	6	ND
Urine gravity	1.02	1.026	ND
Urine β2-microglobulin (μg/L)	359	353	ND
Urine NAG (IU/L)	6	5.2	ND

*AST* aspartate aminotransferase, *ALT* alanine aminotransferase, *GFR* estimated glomerular filtration rate, *RBC* red blood cell, *WBC* white blood cell, *ND* no data, *HPF* high power field, *NAG* N-acetyl-beta-d-glycosaminidase.

Based on the family history, the probands were suspected of having FRG. Genomic DNA was extracted from blood samples from both patients for diagnosis and sequenced using next-generation sequencing (NGS) with a custom panel named SPEEDI-KID^5^ after obtaining written informed consent from the children’s parents. This study was conducted in accordance with the Declaration of Helsinki. Permission was obtained from the institution’s ethics committee. Pooled barcoded libraries for NGS were prepared using SureSelect QXT kits (Agilent Technologies, Inc.) according to the manufacturer’s protocol. The prepared libraries were sequenced via SE 150-bp reads using Illumina MiSeq sequencers. SPEEDI-KID covers genetic variants causing FRG, such as SLC5A1, SLC5A2 and SLC2A2 (GLUT2). We also confirmed the presence of variants by Sanger sequencing. PCR primers were designed to flank approximately 200 bp of the final candidate variants, and the target sequences were amplified by PCR. They were then sequenced using Sanger sequencing on a 3130 DNA Analyzer (Applied Biosystems, Tokyo, Japan)^5^.

We found a missense variant, NM_003041.4 (*SLC5A2*): c.1348G>T: p.Gly450Trp, in exon 11. Single-base substitutions were confirmed by Sanger sequencing (Fig. 1b). There were three carriers of the variant, namely, Patient 1, Patient 2, and their father, and no variants were identified in their mother. This variant was present in the children and their father in a heterozygous state. Thus, a correlation between genotype and phenotype was confirmed in the family. Therefore, the genetic form of this variant was suggested to be autosomal dominant and is not registered in gnomAD v3.1.2 (https://gnomad.broadinstitute.org/), ToMMo 8.3 KJPN (https://jmorp.megabank.tohoku.ac.jp/202102/variants/statistics/), the Human Gene Mutation Database (http://www.hgmd.cf.ac.uk/ac/index.php) or ClinVar (https://www.ncbi.nlm.nih.gov/clinvar/). All databases were accessed in June 2022. In silico evaluation tools of SIFT and PROVEAN predicted p.Gly450Trp of *SLC5A2* as deleterious, and Polyphen-2 predicted it as probably damaging. These programs revealed a high pathological significance prediction score, whereas the 2015 ACMG criteria indicated that the variant was of uncertain significance. Variants of uncertain significance are difficult to interpret; thus, their pathological significance cannot be denied^6^. The G450 residue is conserved among human SGLT family members and across SGLT homologs in other species (Fig. 1c). Based on the molecular diagnosis and clinical features of the family members, it is plausible that this novel missense variant of *SLC5A2* contributes to FRG pathogenesis in a compound heterozygous state.

Approximately 90 variants in the SLC5A2 gene have been reported to be associated with FRG from various countries, including the United States and China^3,7^. In most cases of these reports, the variants are homozygous or compound heterozygous for FRG, and obvious racial differences are not reported. In Japan, a heterozygous variant has been reported, and there is no case that carries the same variants as our patients^8^. Missense variants account for loss of function, as with other variants. It has been reported that patients with heterozygous *SLC5A2* variants tend to exhibit relatively milder glucosuria than those with homozygous or compound heterozygous variants^4^. The patients’ urinary glucose levels were mild, which is consistent with the results of previous studies. SGLT2 is expressed in the S1 segment of the early proximal convoluted tubule and plays a critical role in the process of glucose reabsorption with a 1:1 Na + -to-glucose coupling ratio^2^. It has been reported that variants, even on the C-terminal side of SGLT2, could cause a reduction in SGLT2 expression on the cell membrane of renal tubules. The cubilin-amnionless complex is a receptor protein expressed on the luminal cell membrane of proximal tubules, and a deficit of cubilin causes Imerslund–Gräsbeck syndrome. Imerslund–Gräsbeck syndrome characterized by anemia attributed to selective intestinal malabsorption of cobalamin and low-molecular weight proteinuria is thought to develop in homozygotes. However, it has also been reported that heterozygous variants inhibit the glycosylation of cubilin and prevent membrane expression^9^. Similarly, in these cases, this heterozygous variant might cause a decrease in the membrane expression of proximal tubular cells^10^. FRG is an asymptomatic disorder except for glucosuria. Patients with FRG are not affected by severe clinical consequences, and the disorder is considered to be benign. However, by confirming a diagnosis using genetic testing and distinguishing FRG from diabetes, unnecessary testing or treatments can be prevented. In addition, patients with FRG provide an ideal model for identifying and investigating the pathogenic variants of *SLC5A2*. SGLT2 inhibitors have attracted attention as therapeutic agents for type 2 diabetes in recent years^1^, and the analysis of the *SLC5A2* gene and examination of the pathophysiology of FRG could provide useful clues to the progress of diabetes and its treatment.

There are certain limitations to this report. A quantitative test for urinary glucose excretion was not evaluated, especially for the father who did not undergo examinations in our hospital. However, he does undergo these examinations at another hospital every year. Moreover, histological analysis of the kidneys in the probands was not performed. In conclusion, a novel *SLC5A2* variant was identified in a family with familial renal glucosuria. Further investigations are required to clarify the mechanism of SGLT2.

## Data Availability

The relevant data from this Data Report are hosted at the Human Genome Variation Database at 10.6084/m9.figshare.hgv.3258.
